# Upper and lower airway microbiota across infancy and childhood

**DOI:** 10.1038/s41390-025-03942-0

**Published:** 2025-03-12

**Authors:** Ariel J. Hernandez-Leyva, Anne L. Rosen, Christopher P. Tomera, Elaina E. Lin, Elikplim H. Akaho, Allison M. Blatz, William R. Otto, Joey Logan, Lisa R. Young, Rebecca M. Harris, Samantha A. Whiteside, Andrew L. Kau, Audrey R. Odom John

**Affiliations:** 1 Division of Allergy and Immunology, Department of Medicine, Washington University School of Medicine, St. Louis, MO USA; 2 Center for Women’s Infectious Disease Research, Washington University School of Medicine, St. Louis, MO USA; 3 Perelman School of Medicine, University of Pennsylvania, Philadelphia, PA USA; 4 Department of Anesthesiology and Critical Care Medicine, Children’s Hospital of Philadelphia, Philadelphia, PA USA; 5 Division of Infectious Diseases, Department of Pediatrics, Children’s Hospital of Philadelphia, Philadelphia, PA USA; 6 Department of Medicine, John H. Stroger, Jr. Hospital of Cook County, Chicago, IL USA; 7 Division of Critical Care Medicine, Department of Pediatrics, Nemours Children’s Hospital, Wilmington, DE USA; 8 Division of Infectious Disease, Cincinnati Children’s Hospital Medical Center; Department of Pediatrics, University of Cincinnati College of Medicine, Cincinnati Children’s Hospital Medical Center, Cincinnati, OH USA; 9 Department of Biomedical and Health Informatics, Children’s Hospital of Philadelphia, Philadelphia, PA USA; 10 Division of Pulmonary and Sleep Medicine, Department of Pediatrics, Children’s Hospital of Philadelphia, Philadelphia, PA USA; 11 Department of Pathology and Laboratory Medicine, Children’s Hospital of Philadelphia, Philadelphia, PA USA; 12 Department of Molecular Microbiology, Washington University School of Medicine, St. Louis, MO USA

## Abstract

**Background:**

The upper and lower respiratory tracts feature distinct environments and responses affecting microbial colonization but investigating the relationship between them is technically challenging. We aimed to identify relationships between taxa colonizing the nasopharynx and trachea across childhood.

**Methods:**

We employed V4 16S rRNA gene sequencing to profile nasopharyngeal swabs and tracheal aspirates collected from 172 subjects between 20 weeks and 18 years of age. These samples were collected prior to elective procedures over the course of 20 weeks in 2020 from subjects enrolled in a cross-sectional study. After extraction, sequencing, and quality control, we studied the remaining 147 of 172 nasopharyngeal swabs and 95 of 172 tracheal aspirates, including 80 subject-matched pairs of samples.

**Results:**

Sequencing data revealed that the nasopharynx is colonized by few, often highly abundant taxa, while the tracheal aspirates feature greater diversity. The patterns of colonization identified in the nasopharynx correlate with subject age across childhood.

**Conclusion:**

Our data suggests that there are relatively few species that colonize both the nasopharyngeal tract and the trachea. Furthermore, we observe a pattern of change in the nasopharyngeal microbiota that is correlated with age, suggesting a possible developmental progression of the nasopharyngeal microbiota across childhood.

**Impact:**

- The airway microbiota in childhood plays important roles in respiratory health and immune development.
- In this work, we report on paired nasopharyngeal swab and tracheal aspirate samples from a cross-sectional cohort of children from infancy to 18 years.
- We find that the upper and lower airway microbiota are unlikely to share taxa and do not correlate in terms of diversity.
- We show that the composition of the upper airway microbiota is strongly correlated with age, with a stereotypic developmental trajectory during childhood and adolescence.
- Our results inform our understanding of airway microbiota assembly and may be used to predict airway disease in young children.

## Introduction

The microbial communities of the airway establish early in life, develop alongside the host, and are an important regulator of human respiratory health. For example, airway microbiota containing specific, health-associated, commensal microbes has been linked to reduced inflammation during *Pseudomonas aeruginosa* pneumonia in patients with cystic fibrosis.^1^ On the other hand, viral respiratory infections in young children lead to enrichment of *Moraxella*, *Haemophilus*, and *Streptococcus* species in the upper airway that are themselves associated with increased risk for chronic wheeze and later development of asthma.^2,3^ The microbiota of the upper respiratory tract is also thought to play a role in mediating the incidence and severity of many other diseases, including otitis media, rhinosinusitis, and possibly several neurological disorders.^4,5^

The composition of the airway microbiota, and thus its influence on host health, is shaped by both host-intrinsic and external factors.^6^ Host-intrinsic factors such as pH, surface area, and partial pressure of gases vary throughout the respiratory tract and form distinct environments that foster diverse microbial organisms. Additionally, age can influence the composition of the upper airway microbiota, with progressive development in the nasopharyngeal microbiota occurring from infancy through early adulthood.^7^ External factors include birth mode and feeding during infancy, home and school/daycare environment during childhood, and exposure to smoking, antibiotics, and infections throughout adulthood.^6^ These factors play an important role in determining how bacteria are seeded and cultivated at key developmental life stages. Additionally, features such as season, temperature, and humidity continue to shape rapid fluctuations in community composition.^8^ Understanding how these factors play a role in defining the healthy airway microbiota is important to identify deviations towards dysbiosis.

The respiratory tract is conceptually divided into two regions with mucosal surfaces superior to the larynx (including the nares, nasal vault, oronasopharynx, and laryngopharynx) comprising the upper respiratory tract (URT) and those inferior to the larynx (including the trachea, bronchi, and small airways extending to alveoli) comprising the lower respiratory tract (LRT).^9^ Despite anatomical continuity, the URT and LRT exhibit differences in epithelial cell populations, immunological barriers, and environmental phenomena that support distinct ecologies. For example, the microbial communities of the URT colonize in distinct spatial patterns across an otherwise contiguous mucosa in response to the different epithelia and host-intrinsic features.^10,11^ Thus, the primary determinant of URT community composition is fitness to the environmental niche.^12^ On the other hand, the composition of LRT microbial communities is less dependent on biogeography, and the same taxa can be detected throughout, although the most ecologically rich community belongs to the upper trachea.^13^ Microbial community abundance and richness decreases proportional to anatomical depth into the LRT, supporting a model where immigration of taxa by aspiration and emigration of taxa through mucociliary clearance, cough, or phagocytosis by innate immune cells are the primary factors shaping community composition, rather than regional niche differences.^14,15^ Previous work has found that the bacterial composition of the oropharynx was strongly associated with that of the LRT microbiota in adults.^16^ However, the influence of the various other sites of the upper airway on lower airway composition has not been well cataloged, especially during pediatric development.

In this study, we looked at nasopharyngeal swabs and tracheal aspirates from 172 pediatric subjects collected during elective outpatient surgical procedures. These samples were initially collected as part of a study to determine the degree of concordance in the results of reverse-transcriptase polymerase chain reaction assays for SARS-CoV-2 between upper and LRTs.^17,18^ We profiled these samples using V4 16S rRNA gene sequencing to explore the relationship between the host and the microbiota of the nasopharynx and trachea.

## Methods

### Human subjects ethics

Samples were collected in accordance with ethical standards of the institutional review board at The Children’s Hospital of Philadelphia, which approved the study (IRB # 20-017635). Informed consent, including future research use of residual samples, was obtained from guardians and assent, when appropriate, was obtained from subjects.

### Study cohort

The cohort consisted of 183 SARS-CoV-2 RT-PCR-negative subjects from the previously described convenience sample of pediatric subjects under the age of 18 undergoing elective surgical interventions requiring anesthesia and endotracheal intubation at the Children’s Hospital of Philadelphia.^17,18^ Potential subjects were identified on the day of the procedure and patients and families were enrolled and consent was obtained in the pre-operative waiting room. Additional demographic data regarding recorded history of exposure to antibiotics, corticosteroids, or non-corticosteroid immunosuppressants within the Children’s Hospital of Philadelphia healthcare network were obtained from 172 consenting patients by review of the electronic medical record. These data were limited, however, to contact with the Children’s Hospital of Philadelphia healthcare network and may not capture all exposures that subjects received at other institutions. All subjects were enrolled between July 10th and November 24th, 2020. No additional inclusion or exclusion criteria were applied for this study.

Tracheal aspirates were collected by either an anesthesiologist or pulmonologist from subjects undergoing endotracheal intubation (oral route). No more than 5 mL of isotonic crystalloid solution was instilled into the tracheal lumen and immediately aspirated through an endotracheal tube. Nasopharyngeal swabs were collected the same day in advance of the procedure. Nasopharyngeal swabs were collected as per standard COVID-19 testing procedures at the Children’s Hospital of Philadelphia. Briefly, a swab was inserted straight back into a nostril, aiming posteriorly along the floor of the nasal cavity until it reached the posterior wall of the nasopharynx. The swab was rotated gently against nasopharyngeal mucosa for ten to fifteen seconds, then carefully retracted and placed into viral transport medium. All 172 patients with complete demographic information provided nasopharyngeal swabs and 166 of them provided tracheal aspirates. Samples were stored at −20 °C until shipment. Tracheal samples, intact swabs, or negative controls were shipped on dry ice to Washington University in St. Louis, where they were stored at −80 °C until processing.

### Sample processing and sequencing the V4 region of the 16S rRNA gene

Tracheal aspirates and nasopharyngeal swabs for available subjects were extracted using the QIAGEN Microbiome DNA Extraction Kit (QIAGEN CAT#51704) following manufacturer’s directions. Extraction was completed in 17 batches of 20–22 samples with at least one negative control in every batch by the same two individuals. Subjects were randomly assigned to extraction round such that pairs of nasopharyngeal swabs and tracheal aspirates from the same subject were extracted alongside one another. Negative controls included PBS used in extractions or sterile transport buffer used for tracheal aspirates. For tracheal samples, all available volume was used to extract DNA (ranging between 144 µL and 1000 µL with a median of 585 µL of lavage fluid for samples included in the final analysis). After the extraction of all samples was completed, the samples were processed together for V4 16S rDNA sequencing. The V4–V9 regions of the 16S rRNA genes were initially amplified over 20 cycles of PCR using primers 515F and 1492R using a high-fidelity DNA Polymerase (Thermofisher CAT#11304011). Subsequently, the V4 region of the 16S rRNA gene was amplified 30 cycles from 5 μL of pre-amplified DNA using barcoded 515 F and 806 R primers described in Hazan et al.^19^. The concentration of bacterial V4 16S rRNA gene amplicons was quantitated using the Quant-iT dsDNA high-sensitivity kit (Invitrogen Cat# Q32851). Amplicons were sequenced using an Illumina MiSeq instrument with 2 × 250 bp chemistry. FASTQ files, which each represented a different 96-well plate, were further demultiplexed into individual samples and processed as described.^19^ Read depths, organized by sample type are shown in Fig. S1a. Briefly, demultiplexed FASTQ files were trimmed from the 3’ end using the filterAndTrim function of dada2 package in R (truncLen of 200 and 150 base pairs, maxN = 0, maxQ = 0, maxEE = 0, truncQ = 2).^20^ Error rates were learned from the remaining data and dereplicated forward and reverse reads were merged. The composition of amplicon sequence variants (ASVs) in each sample was then inferred using the dada function of dada2 package. ASVs were classified using the assignTaxonomy function of the dada2 package^20^ trained on a custom database.^21^

### Removal of contaminating sequences

The tables of ASVs for each sample type—nasopharyngeal swabs and tracheal aspirates—were then filtered using decontam v1.16.0.^22^ Both the frequency and prevalence models of decontam were used to eliminate contaminating ASVs. The frequency model of decontam uses 16S rRNA abundance measures in samples to predict contaminating sequences. However, qPCR of 16S rRNA directly from extracted DNA samples was unsuccessful in most cases. We investigated two alternative approaches to estimate bacteria abundance in respiratory samples by: (1) measuring V4-16S amplicon DNA after nested PCR (DNA method), or (2) performing qPCR on V4 16S rRNA from samples after the first round of 16S rRNA PCR (which amplified the V4 – V9 regions, as outlined above; QPCR method). Quantitation of control, NPSWB, and TA samples by both methods prior to filtering are shown in Fig. S1b, c, respectively. QPCR values were compared to CT values generated against a standard curve consisting of purified *Haemophilus influenzae* genomic DNA. There was a nonlinear concordance between the concentration of amplicons originally measured from the completed nested PCR (DNA method) and the V4 16S rRNA qPCR of the amplicons (QPCR method) generated by the first round of 16S rRNA amplification (Fig. S1d). These data indicate that the amplicon concentrations from the second round of amplification are monotonically correlated with the DNA concentration values from the first round of amplification (Fig. S1e, Kendall tau: 0.494, *p* < 0.001). To understand how estimates of 16S rRNA present in samples using the DNA method or QPCR method affect the performance of decontam, we compared the results of decontamination using both versions of the method. This analysis could only be performed on samples for which QPCR measures could be obtained (158/172 nasopharyngeal swabs, 133/166 tracheal aspirates, 34/38 negative control samples).

We suspected that the prevalence and abundance of contaminating ASVs were likely to be dependent on sampling site, so we examined these sites separately.^23^ For the nasopharyngeal swabs, the QPCR method was slightly more sensitive, identifying 7 more contaminants than the DNA method, however every contaminant identified by the DNA method is corroborated by the prevalence model (Fig. S1f). For the tracheal aspirate samples, there is a single ASV difference that the DNA method of the frequency model identifies as a contaminant, but neither the prevalence model nor the QPCR version of the model identify (Fig. S1g). Overall, there were few differences between using the DNA method and QPCR method to input into the frequency decontamination model and we proceeded with the DNA-based method since more samples could be included in the final analysis.

As noted above, contaminating ASVs were likely to be dependent on sampling site, so thresholds for contaminant prediction for the frequency model (Fig. S2a, b) and the prevalence model (Fig. S2c, d) were estimated separately for nasopharyngeal swabs and tracheal aspirates based on the distributions of scores predicted for each model. For the frequency model, bacterial abundance was estimated using the DNA concentration of V4 16S rRNA amplicons after nested PCR (DNA method, see above), consistent with methods used in previously published reports of the airway microbiome.^23^ ASVs classified as a contaminant by either model were removed from the respective set of samples (281 and 433 contaminating ASVs were removed from nasopharyngeal and tracheal aspirate samples, respectively). After decontamination, 147 of 172 nasopharyngeal samples still had greater than 1000 reads and were retained for subsequent analysis (Fig. S2e, g). Of the 166 tracheal aspirates extracted and sequenced, 95 still had greater than 1000 reads after decontamination and were retained for subsequent analysis (Fig. S2f, g). Analysis was performed on samples that were provided with at least 100 μL of volume and retained at least 1000 reads after decontamination.

### Statistical analysis

Statistics and all data analyses were performed in R version 4.2.1. Data are represented as medians with min–max ranges unless otherwise specified. Nonparametric Wilcoxon signed-ranks, Kruskal-Wallis, and Kendall Tau tests conducted for statistical significance are described in the text and figure legends. Linear regression was performed using the lm function in R and generalized linear models were performed using the glm function in R. Where applicable, age and seasonality (week of sample collection) were addressed as continuous variables while all other demographic features were addressed as categorical. Adjustment for *p*-values was performed using Benjamini-Hochberg correction when necessary. Violin plots and box plots display the IQR and the whiskers display 1.5* the IQR. The following symbols are used to denote significance: *p < 0.05, **p < 0.01, ***p < 0.001, ****p < 0.0001. Unifrac distances were calculated using phyloseq v1.46.0 in R. Alpha diversity, and beta dispersion were estimated using vegan v2.4-4 in R. Type III PERMANOVA were conducted using the adonis2 function in vegan with ‘by = “margin”’. Since we were studying two distinct sites, and alpha diversity can be sensitive to differences in sequencing depth^24^ we conducted a sensitivity analysis comparing Shannon diversity against the average Shannon Diversity from 100 iterations of samples rarefied to a count of 1000 reads. Given a strong correlation (Pearson’s correlation R: 0.999987, *p* < 0.0001) between the samples and the rarefied data we elected to carry on analysis by estimating alpha diversity from all the data. Beta dispersion was calculated using the betadisper function from vegan and tested for significance using the anova function in R. When a categorical factor explained a significant variation as determined by PERMANOVA, beta dispersion was assessed. If beta dispersion was not significantly different, it suggested that the centroids of the factor were distinct. If beta dispersion was significantly different we used non-metric dimensional scaling plots generated using “metaMDS” from vegan to assess whether the results of PERMANOVA were associated with dispersion differences alone or both in dispersion and difference in centroid position.^25,26^ Co-occurrence analysis was completed using cooccur v1.3 in R.^27^ Dirichlet multinomial distributions were modeled using the DirichletMultinomial v1.38.0 and the microbiome v1.18.0 packages in R. BLAST searches against the 16S ribosomal RNA sequences database were completed using the online BLASTn software.^28^

## Results

### Description of cohort

The samples from 172 pediatric surgical patients recruited from the Children’s Hospital of Philadelphia were investigated in the study (Table S1). The median age of subjects was 6.6 years (0.13–18 years) of which 106 were male and 66 were female. Matched tracheal aspirate and nasopharyngeal swab samples were available for 157/172 subjects. Twelve different procedure categories were identified, with the most common being ear-nose-throat procedures representing 30.2% of subjects. All samples from subjects were collected during a 20-week span of time (July 10th, 2022–November 24th, 2022). At the time, the Philadelphia, PA metro area was under COVID-19 mitigation, including masking and distancing, and the incidence of common respiratory viral infections was low.

### Decontamination and filtering of low microbial abundance samples from nasopharyngeal swabs and tracheal aspirates

To understand the relationship between the nasopharynx and trachea, we profiled the microbial composition of the nasopharyngeal swabs and tracheal aspirates collected from children under the age of 18 years by sequencing the V4 region of the 16S rRNA gene.^29^ Both of these sample types are generally of low microbial abundance in healthy subjects, which present challenges for analysis because contaminants from reagents or the environment make up a proportionally larger fraction of reads and artificially inflate classic metrics of microbial diversity such as alpha diversity.^6,30^ Several methods have been proposed to identify and remove contaminating sequences from metagenomic data generated from low microbial abundance samples.^31^ Here, we filtered contaminant sequences from ASVs generated by DADA2 with the R package decontam, which models the likelihood of an ASV being a contaminant based on its rate of detection in negative control samples (prevalence model) and the relationship between its abundance and the original DNA concentration of the sample (frequency model; see “methods” for further details).^22^

After decontamination, we used PERMANOVA to identify whether biological differences were still captured by the data (Fig. S2h). Prior to decontamination, sample type was the most important factor (PERMANOVA *R*^2^: 12.5%, *p* < 0.001), however extraction round explained a significant proportion of the variance amongst the samples (PERMANOVA *R*^2^: 8.5%, *p* < 0.001). Batch effect associated with extraction round was anticipated, and so we ensured negative controls were included with every set of 21–22 extracted samples. We observed that the composition of the negative controls also varied with extraction round suggesting a significant effect of the “kit-ome” on our negative controls (Fig. S3).^32^ After decontamination, sample type remained the most important factor (PERMANOVA *R*^2^: 15.3%, *p* < 0.001%) but the effect of extraction round was reduced (PERMANOVA *R*^2^: 6.2%, *p* = 0.077), suggesting our filtering approach successfully reduced contaminating sequences. Ultimately, data from 147 nasopharyngeal swabs and 95 tracheal aspirates were included in the final analysis (Table S1). Of these, 80 nasopharyngeal swabs and tracheal aspirates represented paired samples collected from the same subject. As expected, the demographics between all the remaining nasopharyngeal swab samples and all remaining tracheal aspirate samples are largely well-matched and unremarkable, although samples from subjects that underwent pulmonary bronchoscopic procedures and/or gastrointestinal endoscopy are only represented among the nasopharyngeal swabs. To our knowledge, these data represent one of the largest reported cohorts of matched nasopharyngeal and tracheal samples in children and offer a unique opportunity to explore the microbial composition of the pediatric nasopharynx and trachea.

### Overview of the V4 16S rRNA gene profiles from nasopharyngeal swabs and tracheal aspirates of pediatric patients

**Fig. 1:**
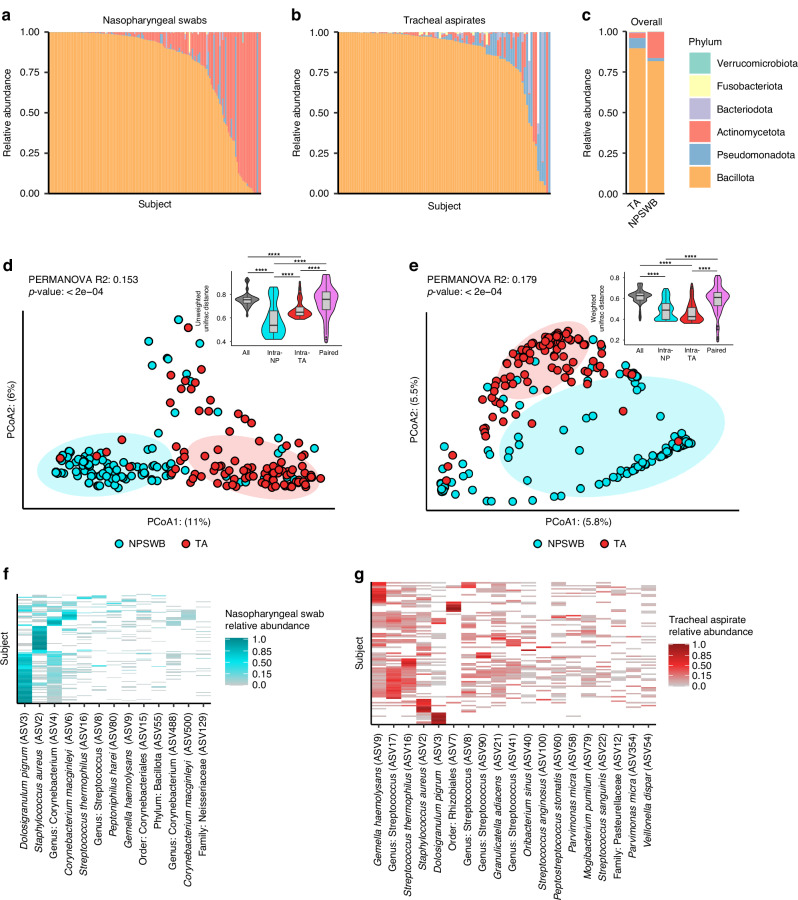
Summary of V4 16S rDNA Sequencing results from upper and lower airway samples. Taxonomic relative abundance plots depicting phyla level abundances for each sample in either the nasopharyngeal swabs (**a**: *N* = 146) or tracheal aspirates (**b**: *N* = 95). White bars reflect the proportion of taxa that were not classified to the phyla level. **c** Summary relative abundance plot showing mean phyla level abundances in nasopharyngeal and tracheal samples. Principal coordinates analysis (PCoA) depicting the unweighted (**d**) and weighted (**e**) Unifrac distances between all samples. Results of PERMANOVA reflect the difference between nasopharyngeal swab and tracheal aspirate microbiomes. Inset: Comparison of Unifrac distances of samples acquired from different individuals at the same site (intra-NP and intra-RTA) to samples acquired from the same individual across anatomic sites (Paired). **f** Heatmap reflecting taxa detected in at least 10% the nasopharyngeal swabs samples. **g** Heatmap reflecting taxa detected in at least 10% of the tracheal aspirate samples.

To review the results of V4 16S rRNA gene sequencing we visualized the data both summarized to the phyla level, and at the level of individual ASVs. Both the nasopharyngeal and tracheal microbiota are occupied by six major phyla: Bacillota (formerly the phyla Firmicutes), Actinomycetota, Pseudomonadota (formerly the phyla Proteobacteria), Bacteriodota, Fusobacteriota, and Verrucomicrobiota (Fig. 1a, b). The most common phylum in both types of samples is Bacillota; however, the second most common phylum varies between the nasopharyngeal swabs and the tracheal aspirates (Fig. 1c). In the nasopharyngeal swab samples, ASVs are more frequently classified as Actinomycetota, whereas in tracheal aspirate samples ASVs are more frequently classified as Pseudomonadota. These overarching differences in community structure are apparent by beta-diversity analysis, where, despite differences in dispersion, nasopharyngeal swab microbiota cluster separately from tracheal aspirate microbiota (Fig. 1d, PERMANOVA *R*^2^: 0.153, *p* < 0.0002, PERMDISP *p* < 0.002). We also see similar results when using a weighted metric, implying that our findings are not dependent solely on presence or absence of microbes (Fig. 1e, PERMANOVA *R*^2^: 0.179, *p* < 0.0002, PERMDISP *p* < 0.0022). We additionally observed that samples acquired from the same site (either NP or TA) were more similar to one another than samples acquired from the same individual at different sites (Figs. 1d, e inset). A more granular inspection of the phylogenetic composition of the nasopharyngeal microbiota reveals that most samples feature a high abundance of *Dolosigranulum pigrum* or *Staphylococcus aureus* (Fig. 1f). The next most abundant taxon tends to be the Actinomycetota *Corynebacterium*. Overall, we observe that the nasopharyngeal swab microbiota is dominated by a few key taxa. Among the most abundant taxa identified in tracheal aspirate microbiota are *Gemella haemolysans* and *Streptococcus* species (Fig. 1g). While *D. pigrum* and *S. aureus* can be detected at high abundance in some samples, most subjects tracheal aspirate microbiota feature numerous taxa with a more even distribution of abundances. While our ability to resolve the taxonomic composition is limited by marker gene sequencing, our data highlights marked differences between the microbiota of the nasopharyngeal swabs and tracheal aspirates.

**Fig. 2:**
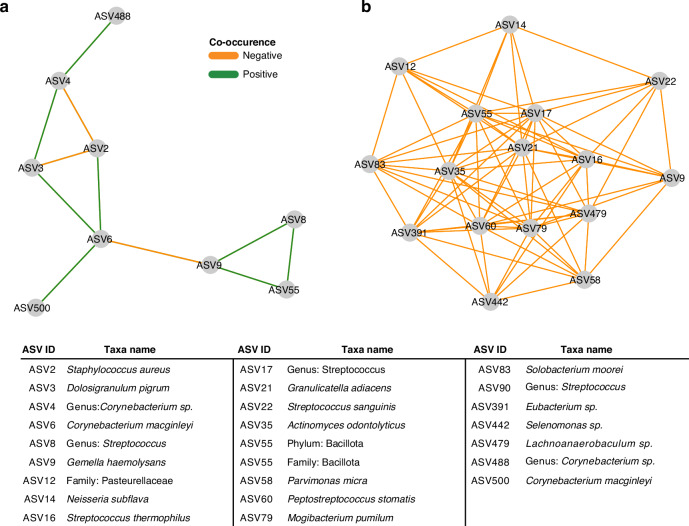
Co-occurrence networks reveal interactions in nasopharyngeal swabs and tracheal aspirate microbiomes. **a** Co-occurrence network generated from V4 16S rRNA profiling of the nasopharyngeal samples. Nodes were only included if they shared an edge with a minimum effect size of 0.05. **b** Co-occurrence network generated from V4 16S rRNA profiling of the tracheal aspirate samples. Nodes were only included if they shared an edge with a minimum effect of 0.10.

To further understand the microbiota of the nasopharyngeal swabs and tracheal aspirates, we conducted a co-occurrence analysis to identify sets of taxa within each community that may share an ecological relationship. Co-occurrence represents the likelihood that two taxa can be identified within the same sample. In our analysis, a positive co-occurrence was defined as a rate of co-occurrence greater than would be expected by chance alone, and a negative co-occurrence represents a rate of co-occurrence less than what would be expected by chance alone. Unusual rates of co-occurrence may represent underlying ecological relationships in an environment.^27,33^ We visualized this analysis as networks within the nasopharyngeal swab microbiota (Fig. 2a) and tracheal aspirate microbiota (Fig. 2b). Among the nasopharyngeal microbiota, we identified a few relationships among the most abundant taxa including a positive co-occurrence between *D. pigrum* (ASV3) and *Corynebacterium* (ASV4) species that has previously been reported in the literature.^34^ Notably, both species also negatively co-occur with *S. aureus* (ASV2), another phenomenon that has been previously described.^34^ We also find a network of three bacteria (*G. haemolysans, Streptococcus*, and an unclassified Bacillota) that comprise another network with an unusually low rate of co-occurrence with *C. macginleyi* (ASV6). There is a greater number of co-occurring taxa in the tracheal aspirate microbiota. Many of these relationships are positive associations between ASVs attributed to *Streptococcus*, consistent with previous reports of lower airway *Streptococcus* colonization linked to aspiration of microorganisms colonizing the oropharynx.^14,35^ Overall, we find the composition of the nasopharyngeal and tracheal microbiota to be consistent with prior findings.

### Defined clusters of taxa describe the nasopharyngeal and tracheal microbiota

**Fig. 3:**
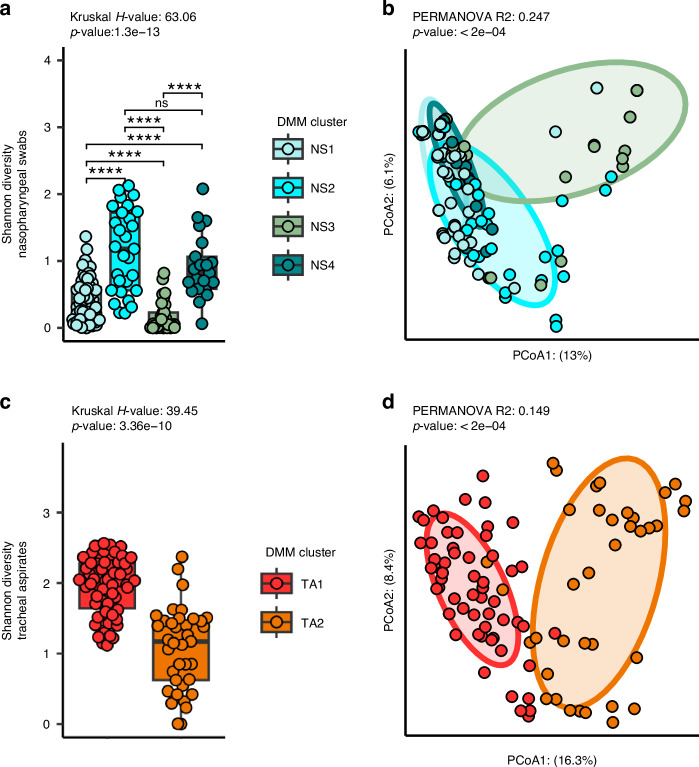
Summary of clusters identified by Dirichlet multinomial modeling of the nasopharyngeal swab microbiomes and tracheal aspirate microbiomes. **a** A comparison of Shannon’s diversity measures for the microbiota of the nasopharyngeal swabs separated by assigned cluster. Nasopharyngeal swabs are divided into four clusters: NS1 (*N* = 68), NS2 (*N* = 29), NS3 (*N* = 29), and NS4 (*N* = 21). **b** Principal coordinates analysis (PCoA) of the unweighted Unifrac distances between nasopharyngeal microbiomes. **c** A comparison of Shannon’s diversity measures for the microbiota of the tracheal aspirates separated by assigned cluster. Tracheal aspirates are divided into two clusters: TA1 (*N* = 58) and TA2 (*N* = 37). **d** Principal coordinates analysis (PCoA) of the unweighted Unifrac distances between tracheal aspirate microbiomes. Kruskal-Wallis test was used to test whether the clusters explained a significant proportion of variance in the alpha diversity of subjects. Wilcox-tests with Benjamini-Hochberg false discovery rate correction was used to identify between-cluster differences. PERMANOVA was conducted to test whether the assigned clusters explained a significant proportion of the variance in the data.

Since we observed several defined networks of taxa within the co-occurrence analysis, we anticipated that the nasopharyngeal and tracheal microbiota of our cohort could be described by a few distinct configurations. Therefore, we employed Dirichlet multinomial modeling to infer clusters from the nasopharyngeal and tracheal microbiota separately. We found that the nasopharyngeal microbiota grouped best into four distinct clusters (Fig. S4a). The cluster NS1 was defined by a higher abundance of *D. pigrum* and *Corynebacterium* compared to other nasopharyngeal swab clusters (Fig. S4b, c). Subjects assigned NS2 featured more diverse and even communities that other nasopharyngeal swab clusters. Subjects assigned NS3 had nasopharyngeal swab microbiota dominated by *S. aureus* with relatively low Shannon’s diversity otherwise. NS4 represented subjects colonized with similar amounts of *S. aureus* and *D. pigrum* but mostly dominated by *Corynebacterium macginleyi*. Overall, we found that the clusters explained a significant proportion of the variation in alpha diversity between subjects (Fig. 3a, Kruskal-Wallis *p* < 0.0001), with NS2 displaying the greatest alpha diversity on average. We also used PERMANOVA to estimate the variation (Fig. 3b, PERMANOVA *R*^2^: 0.247, *p* < 0.0001, PERMDISP *p* < 0.0001) in the nasopharyngeal microbiota that could be attributed to these clusters.

We also found that our tracheal aspirate microbiota grouped best into two clusters (Fig. S5a). TA1, featured high rates of colonization with many different taxa, but especially *Streptococcus, Gemella haemolysans, S. aureus* as well as *Mogibacterium pumilum, Actinomyces odontolyticus*, and *Solobacterium moorei* (Fig. S5b, c). TA2 communities tended to demonstrate a more even community composition that were not dominated by a single taxon. As expected, several of these clusters contain relationships observed in the co-occurrence networks. For example, NS1 seems to represent the positive co-occurrence between *D. pigrum* (ASV3) and *Corynebacterium* ASVs (ASVs 4 and 6). As among the nasopharyngeal microbiota, we also found that the tracheal aspirate clusters explained a significant proportion of the variance in Shannon’s diversity, (Fig. 3c, Kruskal-Wallis *p* < 0.0001) with TA1 featuring the greatest Shannon’s diversity on average, and TA2 featuring the least. The assigned clusters also explained a significant proportion of the variance in the beta-diversity between the tracheal aspirate microbiota (Fig. 3d, PERMANOVA *R*^2^: 0.149, *p* < 0.0002, PERMDISP *p*-value: 0.031).

We evaluated whether any of the clusters were associated with subject demographics. We observed a strong correlation between the nasopharyngeal clusters and age (Table S2, Table S5, Kruskal-Wallis *p* < 0.001). NS1 represents the youngest samples (median age 4.4 years), followed by NS2 (median age 5.64 years), NS3 (median age 9.84 years), and NS4 (14.17 years). On the other hand, there were no notable associations between demographic factors and assigned tracheal aspirate cluster (Table S3, Table S5).

### Diversity of the nasopharyngeal microbiota does not correlate with the diversity of the tracheal microbiota

**Fig. 4:**
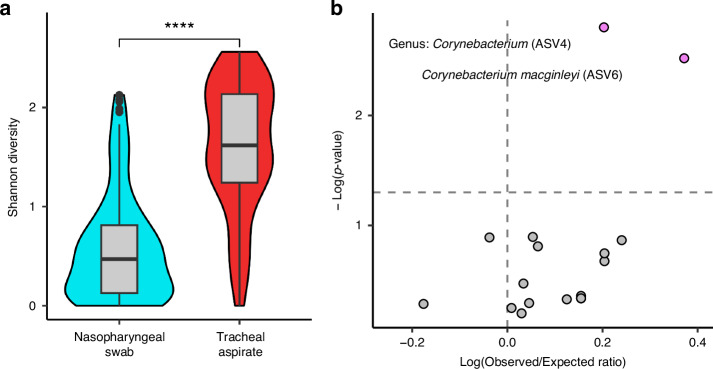
Correlating features of the nasopharyngeal swabs and tracheal aspirates. **a** Shannon’s diversity comparison between nasopharyngeal swabs (*N* = 147) and tracheal aspirates (*N* = 95) using Wilcoxon signed ranks test. **b** Co-occurrence analysis of taxa occurring in both the nasopharyngeal swab and tracheal aspirate of the 80 subjects with matched samples.

We compared the microbial diversity of the nasopharyngeal swab microbiota against the diversity of the tracheal aspirate microbiota using Shannon’s diversity (Fig. 4a). We observed that the tracheal aspirates have a greater microbial diversity on average than the nasopharyngeal swabs (Wilcox-Test *p* < 0.0001). Using the 80 matched samples, we measured the correlation in alpha-diversity between nasopharyngeal swabs and the tracheal aspirates and found no significant association (Kendall’s correlation *p*-value: 0.346). Using a Mantel test, which is used to test if two data matrices are correlated, we found that there was no significant relationship between either unweighted (Mantel *p* = 0.785) or weighted (Mantel *p* = 0.184) beta-diversity measures between the 80 matched nasopharyngeal swabs and the tracheal aspirates samples. We also tested whether the clusters assigned to the nasopharyngeal communities were associated with the clusters assigned to the tracheal communities, but we found no significant association between the clusters of the nasopharyngeal swabs and the clusters of the tracheal aspirates (Chi Square *p*-value: 0.7137). We also evaluated whether taxa that could be found in both the nasopharyngeal swabs and tracheal aspirates might co-occur within the same subject. Once again, we leveraged the 80 subject-matched nasopharyngeal and tracheal samples but found that of the seventeen taxa that appear in both anatomical sites, only two ASVs corresponding to *Corynebacterium* significantly co-occur in nasopharynx and trachea of the same subject more than could be expected by chance alone (Fig. 4b, Table S4). An important limitation of this data, however, is that it remains unclear if these co-occurring ASVs are the same strain or represent two different strains with similar V4 16S rRNA sequences present at both sites.

### Age influences the nasopharyngeal swab microbiota across childhood

**Fig. 5:**
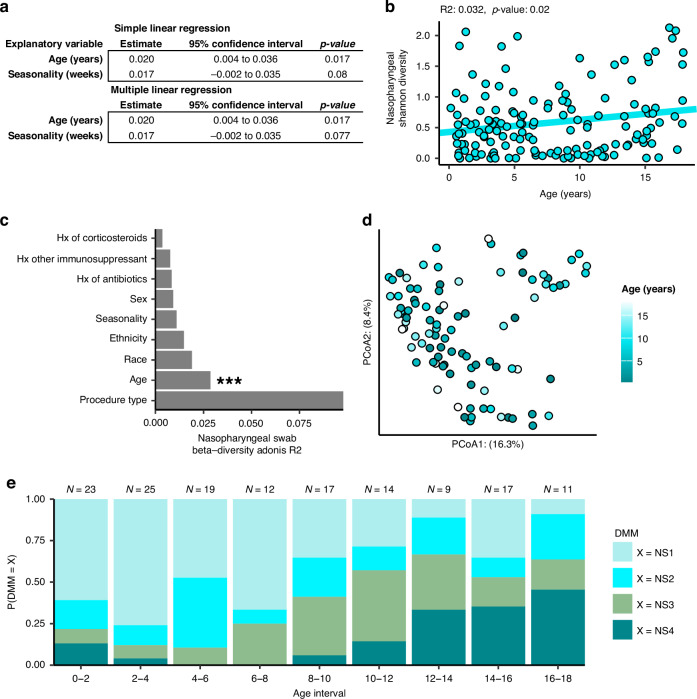
Nasopharyngeal microbiome composition correlates with age across childhood development. **a** Results of linear regression comparing nasopharyngeal swab microbiome Shannon’s diversity against demographic features of the subjects. **b** Plotting the simple linear regression between Shannon’s diversity of nasopharyngeal swabs against subject age. **c** Results of a PERMANOVA analysis comparing unweighted Unifrac distances between subjects against demographic features. **d** Principal coordinates analysis (PCoA) plot rotated to show the influence of age on Unifrac distances between subjects. **e** Bar plot depicting the distribution of nasopharyngeal swab clusters across age. All 147 nasopharyngeal swabs were used in each analysis.

We independently compared the nasopharyngeal swab microbiota and tracheal aspirate microbiota against demographic factors to identify important subject features. We first performed a regression comparing the alpha diversity of nasopharyngeal swabs against the continuous variables of age and seasonality. We found that, while there was no relationship between the Shannon’s diversity of nasopharyngeal samples and seasonality, there was a weak positive relationship between age and nasopharyngeal swab alpha diversity (Fig. 5a, b, *R*^2^: 0.032, *p*-value: 0.02). We also compared nasopharyngeal swab alpha diversity against categorical demographic factors using Kruskal-Wallis tests, but found no associations with race, sex, ethnicity, procedure type, or history of exposure to antibiotics, corticosteroids, or non-corticosteroid immunosuppressants (Table S5). We also investigated the influence of the same demographic factors on the unweighted Unifrac distances between nasopharyngeal swab microbiota and found that age explained a significant proportion of the variance (Fig. 5c,d, PERMANOVA *R*^2^: 0.0310, *p*-value: 0.0014), whereas other covariates did not. Overall, we found that age was robustly associated with nasopharyngeal microbiota diversity and composition, but other factors such as seasonality, procedure type, and history of exposure to antibiotics/corticosteroids were not.

On the other hand, when comparing the alpha diversity of tracheal aspirates against the previously described demographic factors we found no significant relationship with age, seasonality or any categorical demographic variable tested (Fig. S6a, Table S5). We did find an effect of seasonality on the unweighted Unifrac distances between subject tracheal aspirates (Figs. S6b and 6c), PERMANOVA *R*^2^: 0.021, *p*-value: 0.018, PERMDISP *p*-value: 0.006).

Observing an interaction between ecological measures of the nasopharyngeal swab microbiota and age, we next asked whether age might be explained by the clusters predicted by Dirichlet multinomial modeling. Nasopharyngeal swab microbiota cluster was an important predictor of age in a generalized linear model and microbial cluster and age were not independent (Pearson’s chi-square *p* < 0.0001), so we visualized this relationship by looking at the distribution of each cluster across age (Fig. 5e). We observed that the *D. pigrum* dominated NS1 cluster was most common in early childhood from ages of 5 months to 6 years, but that the *S. aureus* dominated NS3 cluster became more common between the ages of 6 to 12. After 12 years of age, the more diverse clusters, NS2, and the *Corynebacterium* enriched communities, NS4, are the most common. Together with the observation that Shannon’s diversity seems to increase over the course of childhood development (Fig. 5b), these findings corroborate age-associated changes in community composition seen in the nasopharyngeal swab microbiota.

## Discussion

In this study we extracted, sequenced, and performed quality control on the nasopharyngeal swabs and tracheal aspirates of over 100 pediatric subjects. This unique cohort containing 80 subject-matched nasopharyngeal and tracheal samples granted us the opportunity to investigate the relationship between the nasopharyngeal and tracheal microbiota, as well as explore the influence of age on the respiratory tract microbiota over the course of childhood development. Our study confirms the reported taxonomic composition of the nasopharynx and trachea found in previous literature,^15,35^ and we identify well-described interactions among the taxa colonizing the nasopharynx. These network relationships were captured by Dirichlet multinomial modeling as distinct clusters. Importantly, we found that the clusters that define the nasopharynx share a strong relationship with age.

We found it surprising that the common metrics of ecologic diversity did not correlate between the nasopharynx and trachea. Moreover, our co-occurrence analysis identified only two ASVs that might be commuting between these two sites, which belong to the same genus of *Corynebacterium*. Other studies have reported evidence of community similarity between different anatomic sites within the respiratory tract such as the anterior nares and nasopharynx.^36^ In contrast, our study had a relatively small number of paired samples using different procedures depending on the site (e.g., swabs for nasopharyngeal specimens vs. aspirates for tracheal samples), and other covariates, such as age, could have reduced our ability to detect similarities between nasopharyngeal and tracheal samples. Nevertheless, our results could support previous assertions that the fitness of most of the taxa colonizing the nasopharynx are sensitive to local environmental conditions and may be poorly adapted to the LRT.^37,38^ Additional, larger studies will be needed to robustly test associations between the upper and lower airway microbiota.

When comparing demographic factors against measures of ecological diversity, we identified an effect of age on both the alpha and beta diversity of the nasopharyngeal swab samples, consistent with recent reports.^7^ We find that the likelihood of an individual harboring a given nasopharyngeal cluster was associated with age. Previous studies of microbial succession of the nasopharynx have reported that healthy infants tend to be colonized by *Corynebacterium* spp. and *D. pigrum*.^37^ This was consistent with our finding that over the first few years of life, infants and toddlers were more likely to be colonized by a community that featured those microorganisms. Beyond early childhood, we saw a shift towards a *S. aureus*-dominated community between the ages of 6 to 12, and then more diverse, mixed community through adolescence. Our findings suggest a stereotypic pattern of development for the microbiota of the nasopharynx defined by increasing community diversity.

Our findings are limited by the cross-sectional nature of the study, limited subject demographic information, relatively small number of paired samples, and the technical constraints inherent to working with samples with low microbial biomass. While we have uncovered an interesting relationship between age and the composition of the nasopharyngeal microbiota, confirming these findings would require dedicated longitudinal study of potential shifts in microbiota composition in individuals over time. As an exploratory study, several comparisons are limited by imbalanced and small group sizes, affecting the power of our statistical analyses. This sample size issue impacts our comparison of microbial communities between the nasopharyngeal and tracheal samples, where a larger number of paired samples may resolve stronger relationships between the two anatomic sites. Additionally, as this study was not originally designed to investigate the microbiota of the respiratory tract and important data that would strengthen our findings, such as vaccination status and a comprehensive history of medication use were not collected. Several of the important external factors such as pollen levels, climate, and pollution were well controlled for in this population, as subjects were enrolled over a window of a few months and in a relatively constrained geographical area, but data about dietary, cultural, or socioeconomic differences were not collected. Moreover, while the normal, healthy human airway is frequently exposed to respiratory viruses, this cohort, collected during the early COVID-19 epidemic, had no recent respiratory infections.

Since all subject in this study were undergoing an elective procedure requiring endotracheal intubation, we also considered if the type of procedure influences the airway microbiota composition. While we found that the airway microbiota was not strongly linked to procedure type, the necessity for an invasive procedure indicates that at least a portion of the study population would not be considered entirely healthy. Procedure types were well distributed, but conditions requiring otolaryngological or maxillofacial procedures may have impacted airway microbiota composition to an unknown extent. Moreover, there are correlations between age of subject and type of procedures. For example, urologic procedures were much more common in the first two years of life (Tables S6–S8). To help demonstrate that our finding of an association between age and the community type of the nasopharynx was not merely a product of procedure type, we repeated the analysis using only subjects from the ear-nose-and-throat procedure group (*N* = 48, age = 6.2 ± 4.8 years) and confirmed that age was still a significant predictor of community type in a generalized linear model (Pearson’s chi-square *p* < 0.0001; see Fig. S7 and Table S5). Despite the complexity of this sample population, we believe that these samples are an important window into the association between the respiratory microbiota and human health and that the results generated here support further investigations in more focused studies.

We faced a final limitation in that nasopharyngeal swabs and tracheal aspirates represent low abundance microbial samples amplified by V4-16S rRNA sequencing that are prone to contamination and bias. Our approach for defining the phylogeny of sequences assigned taxonomy to the species level when there was sufficient confidence,^39–41^ but there are intrinsic limitations in the use of V4-16S rRNA for taxonomic assignment. Thus, while we have a moderate degree of certainty of the accuracy of the taxonomic assignments, some species could be misidentified. We addressed the constraint of low abundance microbial samples by use of controls at multiple stages during the sample processes and have carefully documented our analytical methods to facilitate replication. Our method for sequencing required an initial PCR amplification cycle to achieve sufficient DNA for adequate V4 16S rRNA gene sequencing, which is known to also increase the sensitivity toward contaminant sequences as well.^42^ Pre-amplification was performed with primers that target a larger region of the 16S rRNA gene sequence encompassing the V4 sequence to reduce the risk of generating contaminating V4 sequences. We also employed decontam to identify and remove likely contaminant sequences by comparing our samples against negative controls run alongside every batch of extracted samples.^38^ While these methods are not guaranteed to completely resolve the challenges of low abundance sequences, we are encouraged by the findings in our data that match and support previous literature and our understanding of airway microbial ecology.

We believe our findings add further evidence to the notion that the nasopharyngeal microbiota is associated with age^7^ and have important implications for understanding airway microbiota development. While the ecological succession of the microbial taxa in the URT has been described through the first two years of life, the continuation of a stereotypic pattern throughout childhood would imply a correlation with the physiological, immunological, and behavioral developments of the individual throughout development. A complete description of this pattern through childhood, such as could be provided by a longitudinal clinical study, would provide a metric for understanding the degree and effects of dysbiosis induced by disturbance and treatment and could be used to predict an individual’s risk for disease. By understanding the natural patterns of ecological succession in the upper airways, we may shepherd airway microbiota development towards beneficial, health-associated patterns with vaccination or probiotics.

## Supplementary information

Supplementary information

Supplementary information

## Data Availability

De-identified participant subject data and R code used for analysis are openly available on Zenodo as of November 1st, 2023 through the DOI: 10.5281/zenodo.10064000. Raw V4 16S rRNA gene sequencing data have been deposited at the European Nucleotide Archive and are publicly available as of October 31st, 2023 under the project accession number PRJEB65487.
