# Supplementary material for: Upper and lower airway microbiota across infancy and childhood
Source: Pediatr Res. 2025 Mar 12;98(4):1449–59. doi: 10.1038/s41390-025-03942-0 (PMC12549332; doi:10.1038/s41390-025-03942-0)
Supplement: Supplementary file 2 — Supplementary information [file 41390_2025_3942_MOESM2_ESM.pdf]

Figure S1

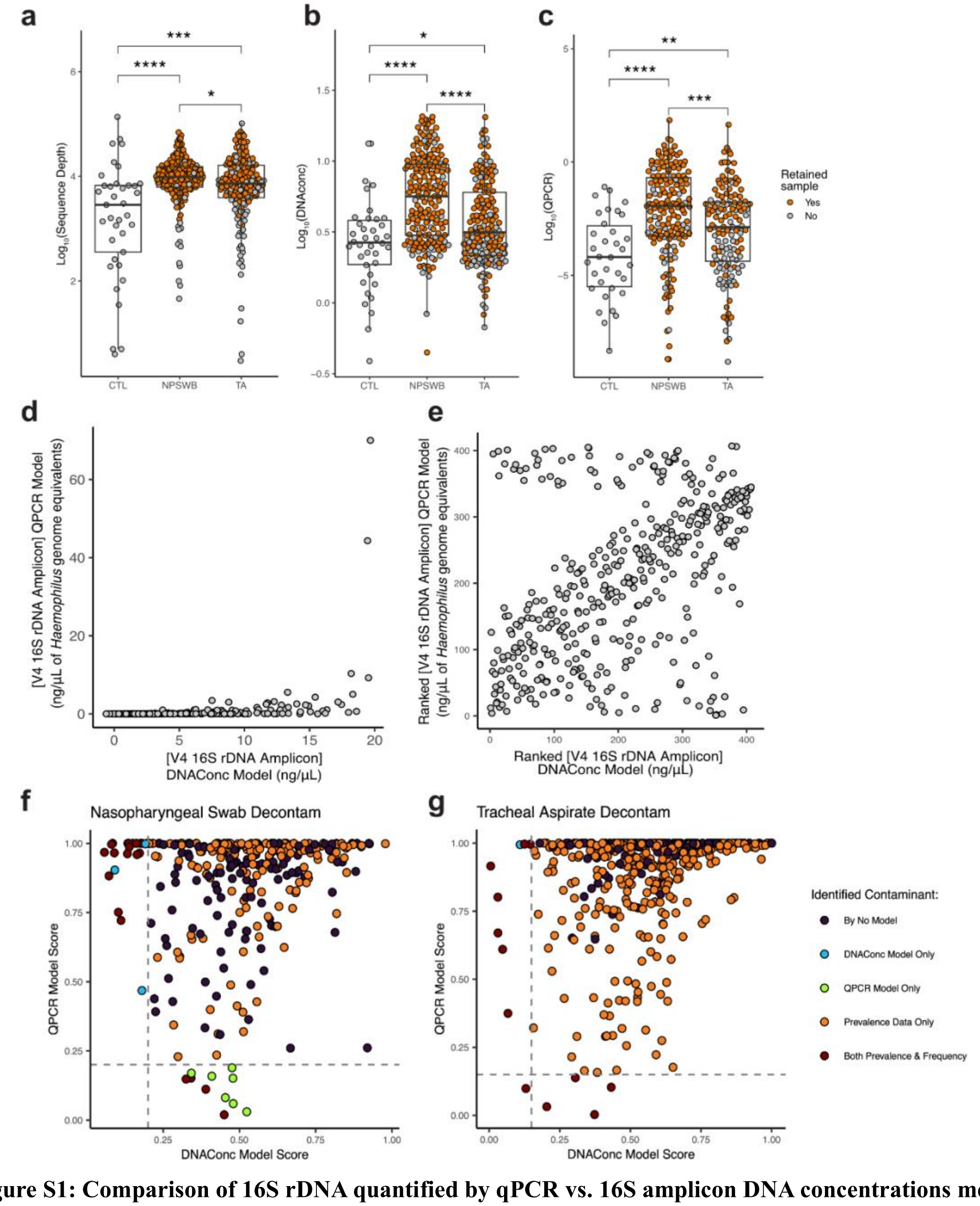

Figure S1: Comparison of 16S rDNA quantified by qPCR vs. 16S amplicon DNA concentrations measured directly after complete nested PCR. Contrasting unfiltered control (CTL), nasopharyngeal swab (NPSWB), and respiratory tracheal aspirate (TA) samples for a) number of reads, b) amplicon DNA concentration after nested PCR, c) QPCR quantitation of V4-16S present in the product of the first nested PCR reaction. Orange points represent samples retained in the analysis outlined in this manuscript. Gray points represent excluded samples. d) Amplicon concentrations estimated by qPCR of the product of the first reaction vs. amplicon concentrations estimated from the product of the second nested PCR reaction. e) The same as d, but comparing the ranks of each. f) Contrasting decontam results from the nasopharyngeal swab data. Each point represents one of 599 ASVs, colored by whether it

was detected as a contaminant and by which model. Orange lines represent the threshold for the respective model to identify a contaminant. g) Same as f, but for tracheal aspirate samples.

Figure S2

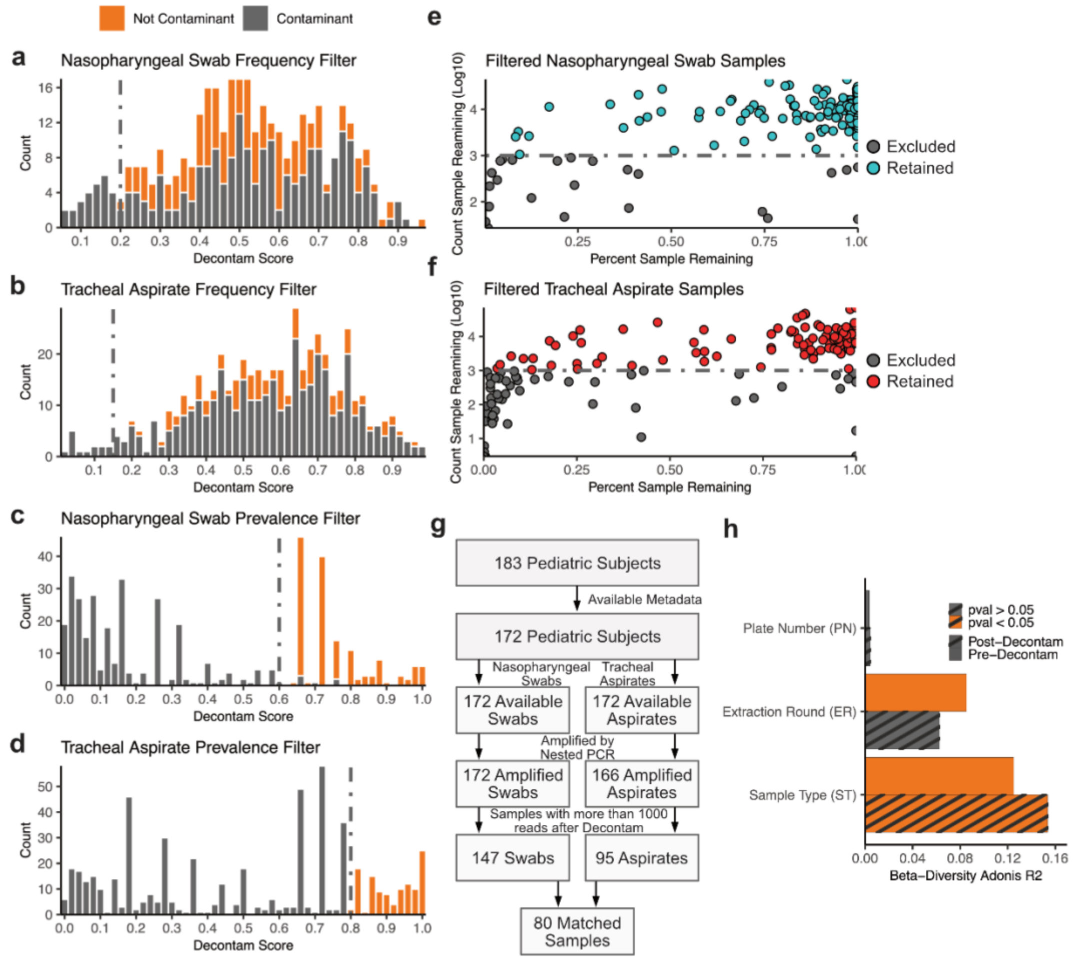

Figure S2: Decontamination and filtering of V4 16S rDNA sequencing of nasopharyngeal swabs and tracheal aspirates. a-b) Histogram of scores generated by decontam fitting the nasopharyngeal swabs and tracheal aspirates to the frequency model for contamination. c-d) Histogram of scores generated by decontam fitting the nasopharyngeal swabs and tracheal aspirates against the prevalence model for contamination. For histograms a-d, dashed, gray vertical lines indicate user-selected thresholds. Gray bars represent counts of ASVs identified as contamination within either nasopharyngeal swabs or tracheal aspirates by either filter. Orange bars represent counts of ASVs that were retained. e-f) Scatter plots showing which nasopharyngeal swab (e) or tracheal aspirate (f) samples were retained vs excluded from further analysis after decontam filtering of ASVs based on the number and percent of reads remaining. g) Flow chart describing subject numbers used in this study. h) Bar plot depicting the results of the PERMANOVA comparing nasopharyngeal and tracheal samples before (solid) and after (striped) filtering with decontam against batch variables and sample type.

Figure S3

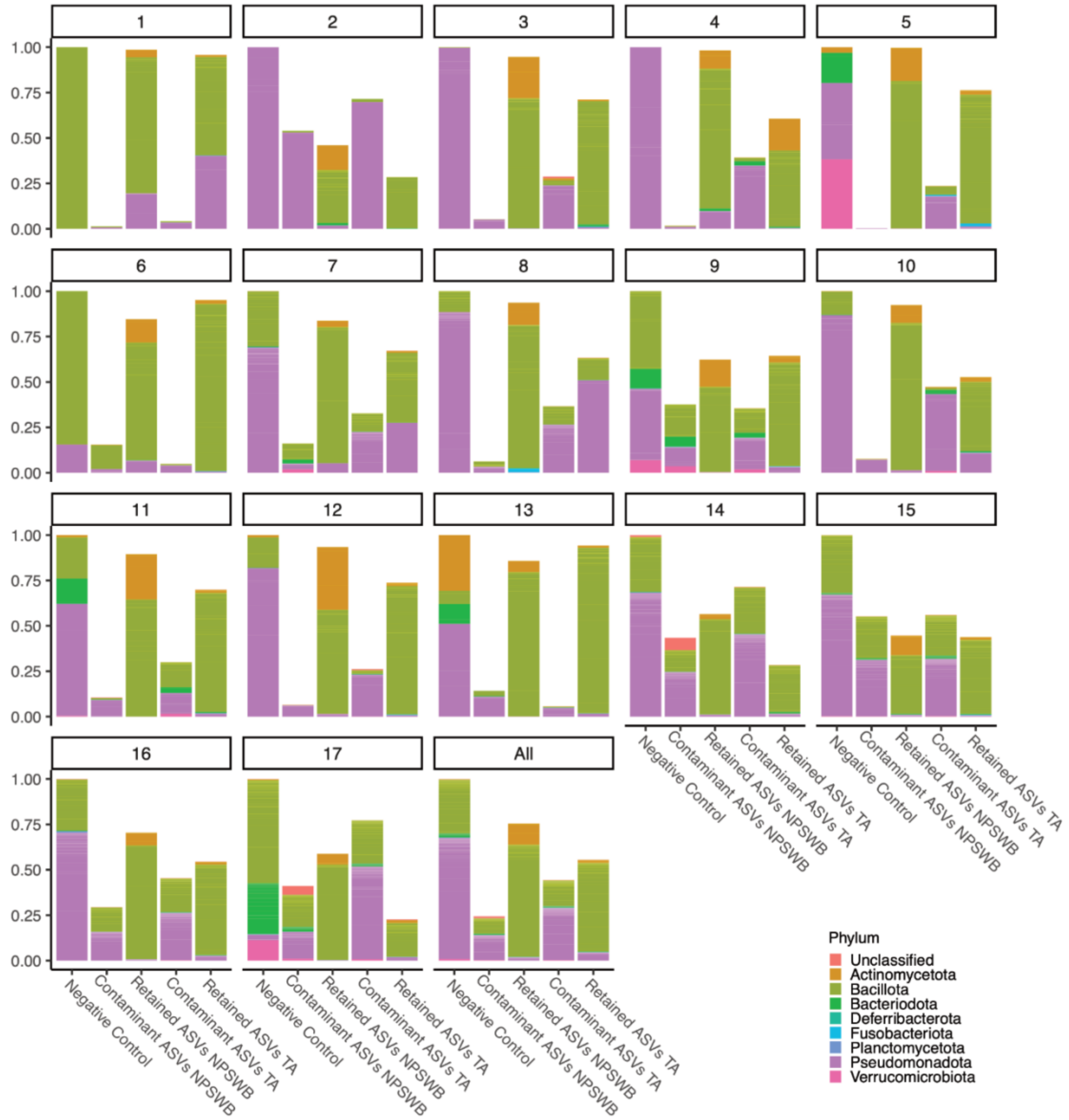

Figure S3: Negative control contaminants are sensitive to kit-associated batch effect. Taxonomic relative abundance microbiome plots describing the composition of contaminant and non-contaminant taxa in each extraction round.

Figure S4

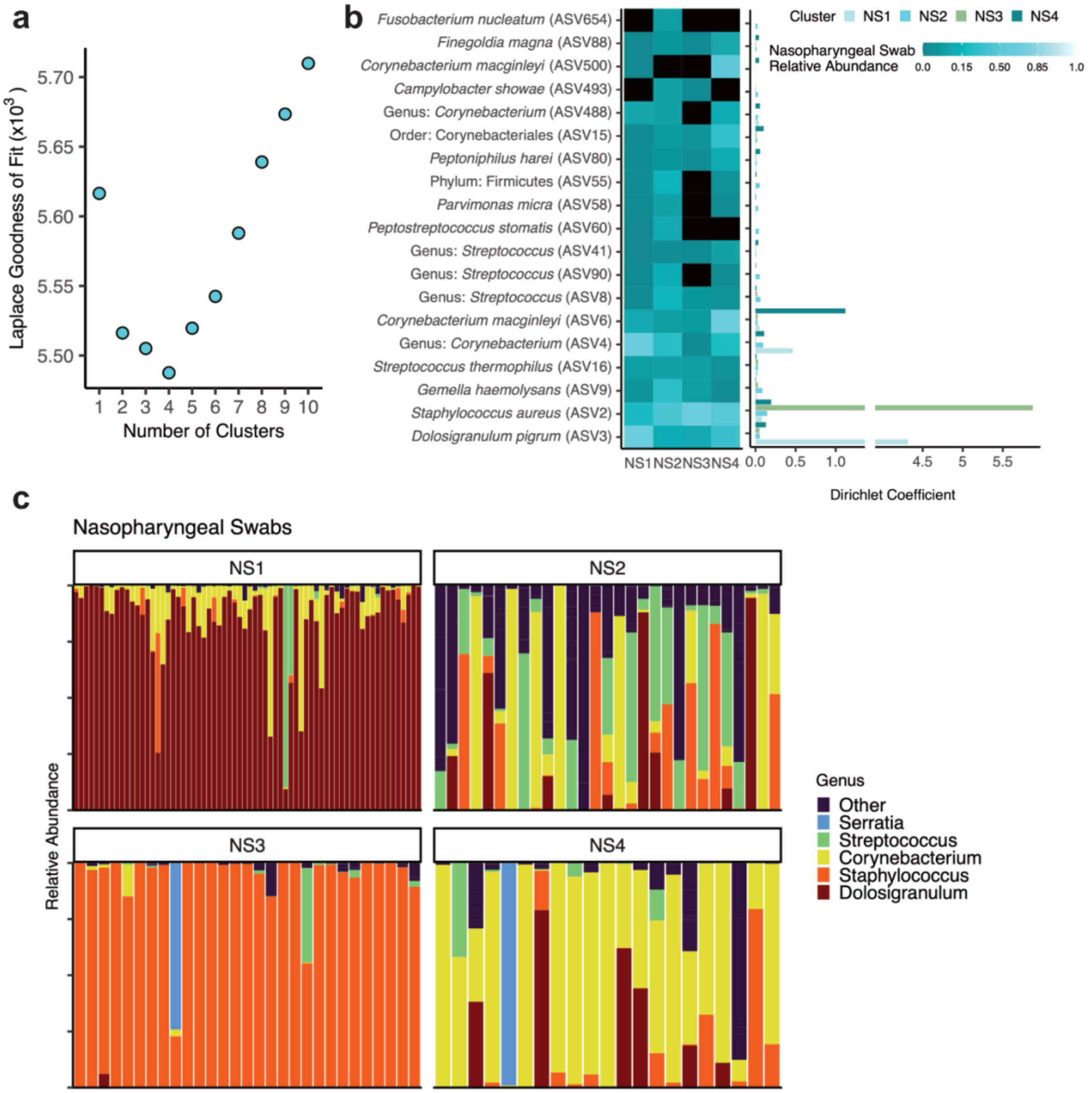

Figure S4: Dirichlet multinomial modeling predicts four distinct community clusters among the nasopharyngeal swabs. a) Laplace goodness of fit comparison for models produced with various numbers of clusters for the nasopharyngeal swabs. b) Heatmaps and bar plots describing the results of Dirichlet multinomial modeling of the nasopharyngeal microbiomes. The heatmaps describe the frequency we detected a particular taxon in subjects separated by DMM cluster. The bar plots describe, for the relevant taxon and for each cluster, the Dirichlet multinomial model coefficient. c) Taxonomic relative abundance plots describing the compositional differences between nasopharyngeal microbiome clusters at the genus level. The top 5 classified genera are indicated separately from other classified taxa.

Figure S5

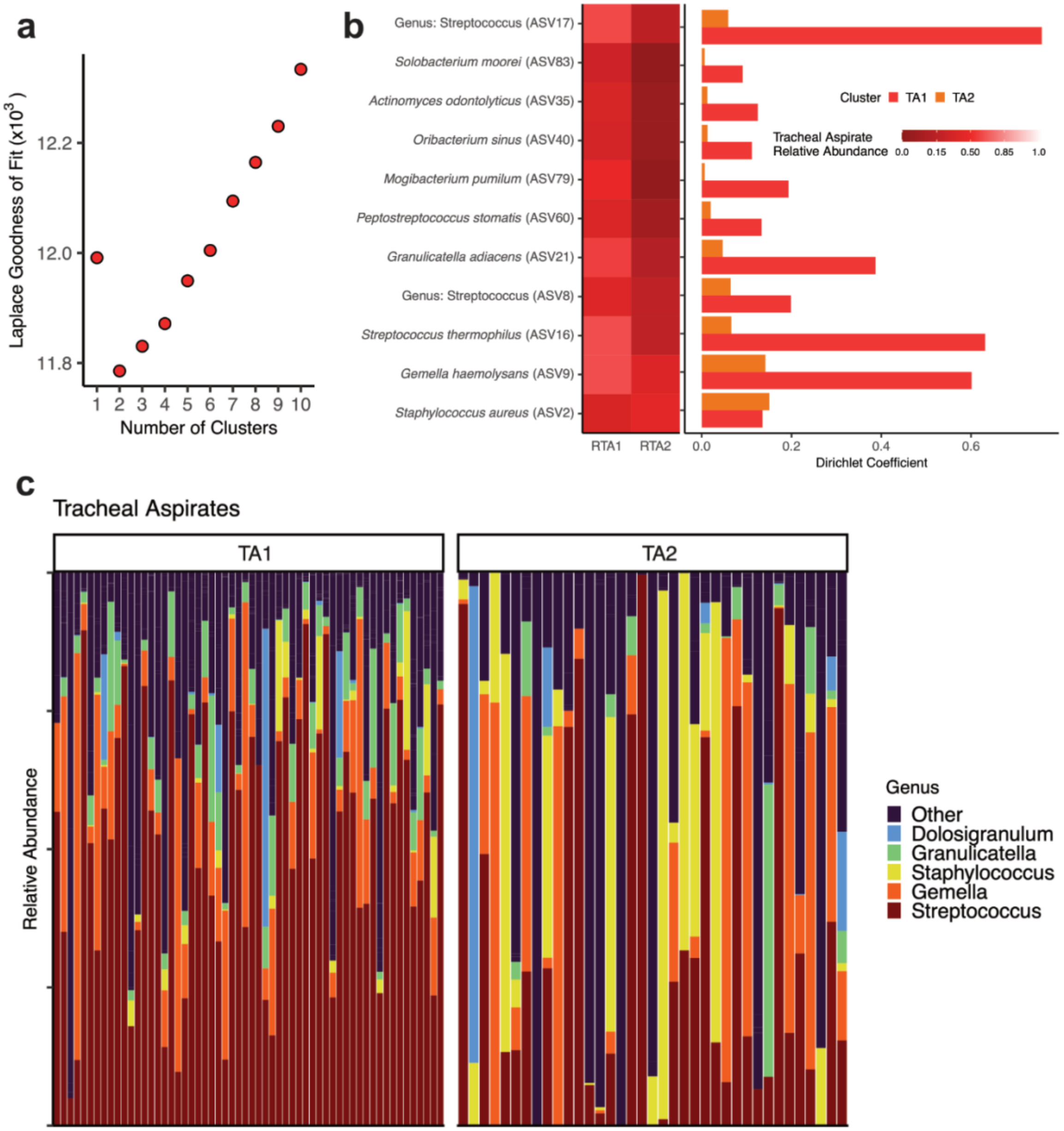

Figure S5: Dirichlet multinomial modeling predicts two distinct community clusters among the tracheal aspirates. a) Laplace goodness of fit comparison for models produced with various numbers of clusters for the tracheal aspirates. b) Heatmaps and bar plots describing the results of Dirichlet multinomial modeling of the tracheal microbiomes. The heatmaps describe the frequency we detected a particular taxon in subjects separated by DMM cluster. The bar plots describe, for the relevant taxon and for each cluster, the Dirichlet multinomial model coefficient. c) Taxonomic relative abundance plots visualizing the compositional differences between tracheal microbiome clusters at the genus level. The top 5 classified genera are indicated separately from other classified taxa.

Figure S6

a

Simple Linear Regression

Explanatory Variable	Estimate	95% Confidence Interval	*p-value*
Age (years)	0.012	-0.012 - 0.037	0.319
Seasonality (weeks)	0.023	-0.005 - 0.050	0.102

Multiple Linear Regression

Explanatory Variable	Estimate	95% Confidence Interval	*p-value*
Age (years)	0.011	-0.013 - 0.036	0.354
Seasonality (weeks)	0.022	-0.005 - 0.049	0.112

b

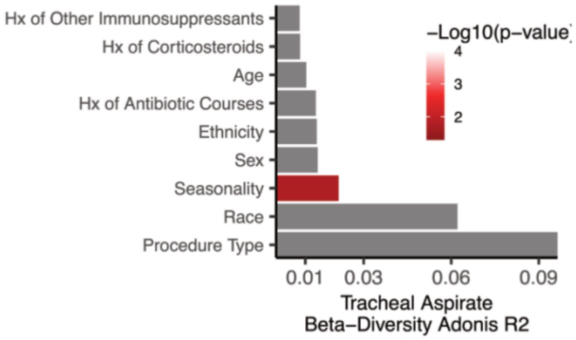

c

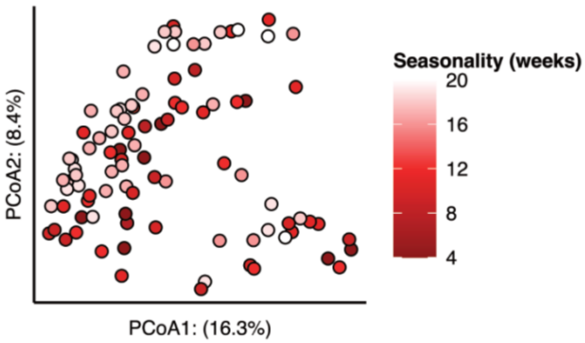

Figure S6: Comparing tracheal aspirate microbiome features against subject demographics. a) Results of linear regression comparing tracheal aspirate microbiome Shannon's diversity against demographic features of the subjects. b) Results of a PERMANOVA analysis comparing unweighted Unifrac distances between subjects against demographic features. c) Principal coordinates analysis showing the influence of age on unweighted Unifrac distances between subject tracheal aspirate microbiomes.

Figure S7

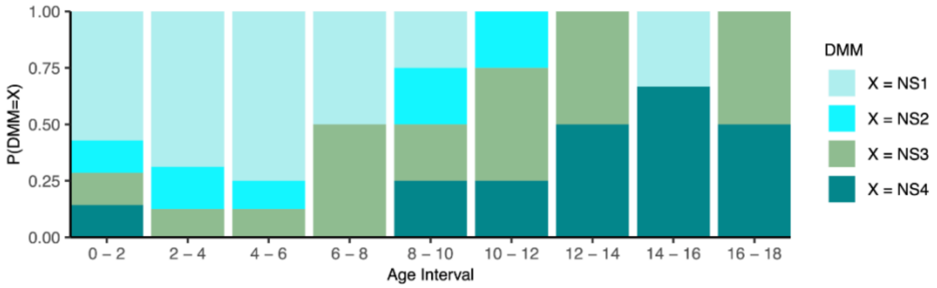

Figure S7 Nasopharyngeal microbiome composition correlates with age across childhood development in a subset of patients undergoing otolaryngologic procedures. Bar plot depicting the distribution of nasopharyngeal Dirichilet microbiome clusters across age. Only 48 nasopharyngeal swabs from subjects undergoing otolaryngologic procedures were used in each analysis (n=48).
